# *In vitro* function, assembly, and interaction of primary cell wall cellulose synthase homotrimers

**DOI:** 10.1101/2024.02.13.580128

**Published:** 2024-02-15

**Authors:** Pallinti Purushotham, Ruoya Ho, Jochen Zimmer

**Affiliations:** Department of Molecular Physiology and Biological Physics, University of Virginia School of Medicine, Charlottesville, VA 22903. Howard Hughes Medical Institute.

## Abstract

Plant cell walls contain a meshwork of cellulose fibers embedded into a matrix of other carbohydrate and non-carbohydrate-based biopolymers. This composite material exhibits extraordinary properties, from stretchable and pliable cell boundaries to solid protective shells. Cellulose, a linear glucose polymer, is synthesized and secreted across the plasma membrane by cellulose synthase (CesA). Plants express several CesA isoforms, with different subsets necessary for primary and secondary cell wall biogenesis. The produced cellulose chains can be organized into fibrillar structures and fibrillogenesis likely requires the supramolecular organization of CesAs into pseudo sixfold symmetric complexes (CSCs). Here, we structurally and functionally characterize a set of soybean (Gm) CesA isoforms implicated in primary cell wall biogenesis. Cryogenic electron microscopy analyses of catalytically active GmCesA1, GmCesA3, and GmCesA6 reveal their assembly into homotrimeric complexes, stabilized by a cytosolic plant conserved region. Contrasting secondary cell wall CesAs, a peripheral position of the C-terminal transmembrane helix creates a large, lipid-exposed lateral opening of the enzymes’ cellulose-conducting transmembrane channels. Co-purification experiments reveal that homotrimers of different CesA isoforms interact *in vitro* and that this interaction is independent of the enzymes’ N-terminal cytosolic domains. Our data suggest that cross-isoform interactions are mediated by the class-specific region, which forms a hook-shaped protrusion of the catalytic domain at the cytosolic water-lipid interface. Further, inter-isoform interactions lead to synergistic catalytic activity, suggesting increased cellulose biosynthesis upon homotrimer interaction. Combined, our structural and biochemical data favor a model by which homotrimers of different CesA isoforms assemble into a microfibril-producing CSC.

## Introduction

Cellulose is a versatile biopolymer and a fundamental building block of plant cell walls, like structural steel in the building industry. It is a linear polymer of glucose units that can be assembled into fibrillar structures. In plant cell walls, cellulose microfibrils are spun around the cell and integrated with a variety of other biopolymers to form the load bearing wall component (Turner and Kumar, 2018).

Cellulose is synthesized by cellulose synthase (CesA), a membrane-integrated processive family-2 glycosyltransferase (GT) (Lombard et al., 2014). Remarkably, CesA synthesizes cellulose from UDP-activated glucose (UDP-Glc) and, during the synthesis reaction, translocates the polymer across the plasma membrane through a channel formed by its own membrane-spanning segment (McNamara et al., 2015). This multi-tasking requires functionally integrating a cytosolic catalytic GT domain with a membrane-spanning secretion channel.

Structural and functional analyses of bacterial and plant CesAs provided detailed insights into cellulose synthesis and secretion (Morgan et al., 2013; Purushotham et al., 2020; Zhang et al., 2021a). Importantly, CesA binds one substrate molecule at a time, transfers a single glucosyl unit to the nascent cellulose chain in an elongation reaction, and, subsequently, translocates the elongated polymer into its channel (Morgan et al., 2016). Cellulose translocation positions the newly added terminal glucosyl unit (the acceptor) for another elongation reaction.

The mechanism of cellulose biosynthesis is evolutionarily conserved, from bacteria to land plants. Accordingly, the architectures of bacterial and plant CesAs share many structural features, including a cytosolic catalytic GT-A domain, an arrangement of three amphipathic interface helices (IF helices) that connect the GT domain with the TM region, and a cellulose secreting TM channel formed from at least six TM helices (Morgan et al., 2013; Purushotham et al., 2020; Zhang et al., 2021a). A defining sequence motif of CesAs and related polysaccharide synthases is the QxxRW motif. The motif’s Trp residue stabilizes the acceptor glucosyl unit right above the catalytic pocket, at the entrance to the TM channel (Morgan et al., 2013).

Plant CesAs contain specific domains absent in most bacterial homologs. These include an extended cytosolic N-terminus beginning with a RING-like region, a plant conserved region (PCR), as well as a class-specific region (CSR) (Carpita, 2011; Purushotham et al., 2020; Wilson et al., 2021). The CSR and PCR are integrated into peripheral extensions of the catalytic domain. Further, plants express different CesA isoforms at different developmental stages, of which certain subsets are necessary for primary and secondary cell wall formation (Persson et al., 2007; Taylor et al., 2003; Turner and Somerville, 1997). Isoforms associated with primary cell wall formation include CesA1, 3 and 6, while secondary cell wall CesA isoforms include CesA4, 7 and 8. The isoenzymes vary the most within the CSR and the N-terminal domain.

Plants organize the individual cellulose polymers into micro and macro-fibrils (Kubicki et al., 2018; Turner and Kumar, 2018). In a fibril, the cellulose polymers are aligned along the fiber axis, leading to exceptionally stable biomaterials. Cellulose microfibrils likely originate from supramolecular CesA complexes (CSCs). CSCs have been identified in different species and, in land plants, appear primarily as pseudo sixfold symmetric membrane-integrated particles by freeze fracture electron microscopy analyses (Herth and Weber, 1984; Kimura et al., 1999; Nixon et al., 2016). The CSC repeat unit likely contains three CesA subunits, thereby accounting for 18 CesAs per CSC and, accordingly, 18 cellulose polymers in a CSC-synthesized microfibril (Nixon et al., 2016; Turner and Kumar, 2018).

Supporting this model, cryogenic electron microscopy (cryo EM) analyses of secondary cell wall hybrid aspen CesA8 and rice CesA7 revealed homotrimeric complexes (Purushotham et al., 2020; Zhang et al., 2021a). Each subunit produces a cellulose polymer and the trimer interface is formed by the PCR and the C-terminal TM helix.

Genetic analyses demonstrated that different CesA isoforms are necessary for proper primary or secondary cell wall formation (Fagard et al., 2000; Turner and Somerville, 1997). Further, co-immunoprecipitation analyses suggested direct interactions between primary or secondary cell wall CesA isoforms (Gonneau et al., 2014; Taylor et al., 2003; Timmers et al., 2009). Combined, these observations nurtured the hypothesis that CesAs organize into heterotrimers of different isoforms *in vivo*, which assemble into CSCs containing six copies of each isoenzyme.

To analyze the oligomerization and function of primary cell wall CesAs, we recombinantly expressed and purified *Glycin max* (soybean) CesA1, CesA3 and CesA6. Cryo EM analyses of all three CesA isoforms reveal the formation of homotrimeric complexes, similar to secondary cell wall CesAs. The primary cell wall CesAs reveal a new position of transmembrane (TM) helix 7, resulting in a cellulose translocation channel with a large lateral exit. All three CesAs are catalytically active *in vitro*, with comparable catalytic rates and pH optima. *In vitro* interaction studies demonstrate that homotrimers of different CesA isoforms interact, leading to synergistic cellulose biosynthesis. Our results support a model by which CSCs consists of homotrimers of different CesA isoforms.

## Results

### Primary cell wall CesAs purify as high and low molecular weight species

**Figure 1.**
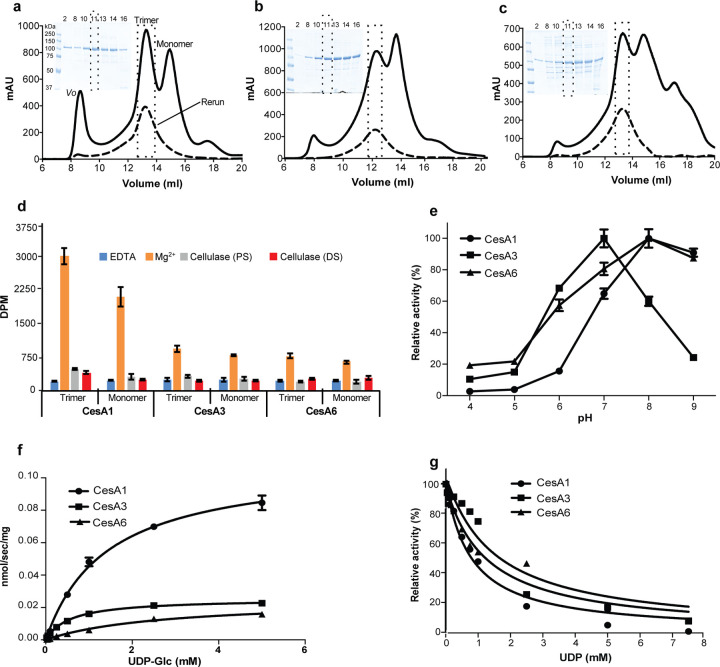
Functional characterization of *Glycine max* primary cell wall CesAs. From **(a-c)** Analytical size exclusion chromatography (Superose 6 Increase) of GmCesA1 **(a)**, GmCesA3 **(b)**, and GmCesA6 **(c)**. Void volume (*Vo*) and trimer and monomer peaks are marked. A rerun of the trimer fraction for each species is shown as a dashed profile. Inset: Coomassie-stained SDS-polyacrylamide gel electrophoresis of the indicated elution volumes. **(d)** Catalytic activity of the purified CesAs. ^3^H-labeled cellulose synthesized by trimeric and monomeric species was degraded with cellulase, followed by quantification by scintillation counting. (DS) and (PS) indicate cellulase treatments during and after the synthesis reaction, respectively. DPM: disintegrations per minute. **(e)** pH optima for catalytic activity of CesA1, 3 and 6. Activities are normalized to the highest activity for each isoform. **(f)** Kinetic analyses of CesAs by titrating UDP-Glc and quantification of the released UDP using an UDP-Glo assay kit. **(g)** UDP inhibits CesAs. Cellulose biosynthesis in the presence of 1.4, 0.5, and 2.3 mM UDP-Glc for GmCesA1, 3, and 6, respectively, and the indicated increasing concentrations of UDP. Product yields in the absence of UDP were set as 100 %. Error bars in panels **d-g** represent deviations from the means of at least three replicas.

To biochemically and structurally characterize soybean primary cell wall CesAs, we followed a similar heterologous expression protocol as previously described for hybrid aspen CesA8 (Purushotham et al., 2020). In short, the CesAs (Figure supplement 1) were expressed with N-terminal poly-histidine tags in SF9 insect cells and purified by metal affinity and size exclusion chromatography in the detergent glyco-diosgenin (GDN) (Materials and Methods). Size exclusion chromatography separated all CesA isoforms into high and low molecular weight species (Figure 1a–c). Cryogenic and negative stain EM analyses identified these species as CesA trimers and monomers, respectively, (Purushotham et al., 2020) (see below).

To test whether micelle-stabilized CesA trimers dissociate into monomers over time, the purified CesA trimers were reinjected onto the size exclusion chromatography column after an overnight incubation on ice. The reinjected material eluted as a trimeric complex, indicating that assembled trimers are stable and do not interconvert with monomers within this timeframe (Figure 1a–c). The co-purifying monomers likely arise from incompletely assembled trimers or oligomer dissociation during membrane solubilization.

### Primary cell wall CesAs are catalytically active *in vitro*

Cellulose biosynthetic activity was quantified by measuring the incorporation of ^3^H-labeled glucose into insoluble cellulose, followed by scintillation counting after removal of excess substrate (see Materials and Methods) (Purushotham et al., 2016). As shown in Figure 1d, the relative activities of the monomeric and trimeric CesA fractions are comparable for each isoform, demonstrating that both species are catalytically active *in vitro*. Between the different isoforms, CesA1 exhibits greatest product accumulation, whereas CesA3 and CesA6 yield about 25% of product, compared to CesA1 (Figure 1d). In all cases, the *in vitro* synthesized polymer is readily degraded by a cellulase, indicating the formation of authentic cellulose. No product is obtained in the presence of EDTA, in agreement with previous observations (Purushotham et al., 2016).

To further assess catalytic differences between the CesA isoforms, we determined their pH optima for catalytic activity and turnover kinetics by titrating the substrate UDP-Glc and quantifying the released UDP in an enzyme coupled reaction (see Materials and Methods) (Figure 1e and f). All CesA isoforms show greatest catalytic activity at neutral pH. CesA3 exhibits an activity optimum at pH 7 with a sharp decline at pH 8 and 9. In contrast, the activities of CesA1 and CesA6 peak at pH 8, with a slight decline at pH 9 (Figure 1e). Further and consistent with the cellulose quantification assay, CesA1 has an apparent Vmax of approximately 0.1 nmol/(sec mg), about four and five times higher compared to CesA3 and CesA6, respectively (Figure 1f). The affinities for substrate binding range from 1.4 mM for CesA1 to 0.6 and 2.4 mM for CesA3 and CesA6, respectively. Lastly, we analyzed inhibition of the isoforms by UDP, which competitively inhibits CesA8 and other related enzymes (Gow and Selitrennikoff, 1984; Omadjela et al., 2013; Purushotham et al., 2016; Tlapak-Simmons et al., 2004). CesA1’s apparent Ki for UDP is roughly 0.8 mM, whereas this concentration is increased to about 1.2 to 1.5 mM for CesA6 and CesA3, respectively (Figure 1g).

### Primary and secondary cell wall CesAs assemble into homotrimers

Cryo EM analyses of the high molecular weight fractions of the purified soybean CesAs (Figure 1a–c) revealed their organization into 3-fold symmetric homotrimers (Figure 2a - figure supplements 2 and 3 and Supplement table 1). For all isoforms, two-dimensional classification identified trimeric particles similar to poplar CesA8 and rice CesA7 (Purushotham et al., 2020; Zhang et al., 2021a). Particle classification in 3 dimensions followed by non-uniform and local refinements generated cryo EM maps ranging in resolution from about 3.0 to 3.3 Å. We applied 3-fold symmetry (C3) during the refinement steps.

**Figure 2.**
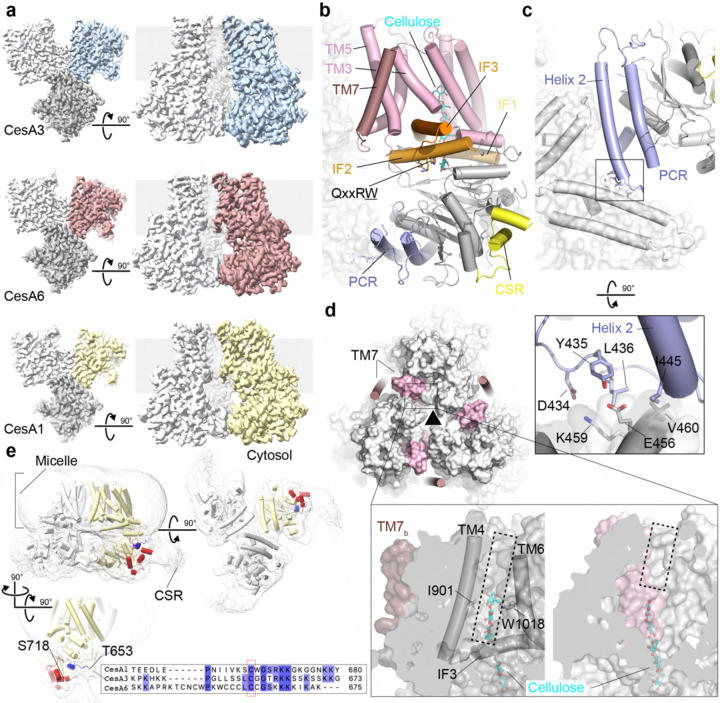
Soybean primary cell wall CesAs assemble into homotrimers. **(a)** CryoEM maps of the CesA homotrimers contoured at 4.5–5.6 σ. One subunit is shown in color, the others are shown in light and dark gray. The gray background indicates the estimated membrane boundaries. **(b)** Cartoon representation of a CesA6 protomer. The transmembrane region is shown in light and dark pink, interface helices (IF) are shown in orange, and the catalytic domain is colored gray. The PCR and CSR regions are shown in blue and yellow, respectively. **(c)** Cytosolic view of the CesA trimer highlighting the interactions of the PCR domains. **(d)** Comparison of CesA6 and CesA8. Soybean CesA6 is shown as a cartoon and overlaid with hybrid aspen CesA8 (surface, PDB: 6WLB). Transmembrane helix 7 is colored light and dark pink for CesA8 and CesA6, respectively. The black triangle indicates the 3-fold symmetry axis of the homotrimer. Zoom views: Surface representations of CesA6 (left) and CesA8 (right) highlighting the lateral window. TM7_b_ refers to TM helix 7 of another protomer. **(e)** CryoEM map of the CesA1 trimer shown at a low contour level (1.4 σ). The CesA1 structure is shown as a cartoon with one protomer colored yellow. The resolved CSR N- and C-terminal regions are colored blue and red, respectively. Bottom panel: Sequence alignment of the CSR regions of CesA1, 3 and 6 generated in Clustal Omega (Larkin et al., 2007).

Overall, the CesAs contain a cytosolic catalytic domain that interacts with the channel-forming TM region via three amphipathic IF helices (Figure 2b). The helices surround the entrance to the TM channel. The Trp residue of the conserved QxxRW motif, located in IF helix 2, sits at the cytosolic channel entrance, right above the catalytic pocket, as previously observed for cellulose, chitin and hyaluronan synthases (Chen et al., 2022; Maloney et al., 2022; Morgan et al., 2013; Ren et al., 2022).

The CesA trimers are stabilized by the PCR domain that is inserted into the catalytic domain’s GT-A fold (Lairson et al., 2008) between β-strands 2 and 3. The PCR contains two anti-parallel α-helices connected by a loop that folds back onto the helical pair (Figure 2c). Within a trimer, conserved residues of the PCR loop contact an equally conserved central region of PCR helix 2 of a neighboring protomer. Specifically, the conserved DYLK motif (residue 434 to 437 in CesA6) of the PCR loop interacts with residues 456 to 460 in PCR helix 2 of the neighboring subunit. These residues belong to the conserved E/DYEEFKVR motif. The interprotomer contacts form a salt bridge between Asp434 and Lys459, a hydrogen bond between Tyr435 and Glu456, and van der Waals interactions between Leu436 and Ile445 with Glu456 in the opposing protomer. All residue numbers refer to CesA6. No differences were observed at this PCR interfaces between the CesA isoforms.

### The PCR trimer forms a positively charged interface

As noted earlier for the secondary cell wall CesAs (Purushotham et al., 2020), the triangular PCR arrangement in an assembled CesA complex positions the side chains of conserved Lys and Arg residues towards the 3-fold symmetry axis. These residues, including Arg449, Lys452, and Arg453 in CesA6, are located in PCR helix 2. Also consistent with previous observations on CesA8 and CesA7, the conserved positively charged residues coordinate an unidentified ligand on the membrane distal and proximal side of the PCR triangle. For all three CesA isoforms, the ligands’ shapes are similar, suggesting that they represent the same small molecule (Figure supplement 3c). On the membrane proximal side, the density extends by about 9 Å towards the membrane, consistent with a nucleotide bound in different poses, as previously suggested (Purushotham et al., 2020).

### TM helix 7 sits at the periphery of the CesA trimer

A notable structural difference between secondary and primary cell wall CesAs is the location of the C-terminal TM helix 7. In hybrid aspen CesA8 and rice CesA7, the helix rests against TM helices 5 and 6 of a neighboring protomer in the trimer, thereby contributing to its TM architecture (Figure 2d). In the soybean primary cell wall CesAs, however, the helix is more flexible, as evidenced by weaker density maps. The helix is shifted to the periphery of the trimer, primarily stabilized by intraprotomer contacts. TM helix 7 has been modeled for CesA3 and CesA6, while its density is detectable but too discontinuous for modeling in CesA1 maps. In the new position, TM helix 7 rests against TM helix 5 and the C-terminal segment of TM helix 3 of the same protomer (Figure 2b and d).

The displacement of TM helix 7 away from the channel architecture of the neighboring subunit creates a large lateral lipid-exposed channel opening (Figure 2d). This window is formed by the N-terminal region of IF helix 3 and TM helices 4 and 6. About midway across the membrane, the opening is roughly 6 Å wide, for example between Ile901 in TM helix 4 and Trp1018 in TM helix 6. This likely exposes the translocating nascent cellulose polymers to the hydrophobic milieu of the lipid bilayer; yet no structured acyl chains (for example from detergent molecules) are observed in this hydrophobic pocket. A similar lipid exposed polysaccharide translocation pathway has recently been described for hyaluronan synthase (Maloney et al., 2022).

### A largely unstructured NTD and CSRs that extend from the CesA trimer’s corners

Although not resolved at high resolution, the N-terminal domain (NTD) of hybrid aspen CesA8 extends as a stalk into the cytosol along the trimer’s symmetry axis (Purushotham et al., 2020). Two-dimensional classification and a low resolution 3-dimensional reconstruction of GmCesA3 reveal a similar cytosolic extension (Figure supplement 2). The NTD is not resolved for GmCesA1 and CesA6, suggesting that it adopts multiple conformations, with the stalk being one of them.

Similarly, except for short helical segments at its N- and C-terminal ends, the CSR is largely disordered in all CesA structures reported to date, consistent with its predicted flexibility (Scavuzzo-Duggan et al., 2018). For GmCesA1, however, at lower contour levels, additional density is observed extending from the corners of the CesA trimer (Figure 2e). Viewed from the cytosol, the extra density resembles a hook extending by about 20 Å clockwise and tangentially along the trimer surface at the water-membrane interface. In this conformation, the CSR’s N-terminal conserved cysteine residue(s) postulated to be acylated (Kumar et al., 2016) is near the membrane surface.

### CesAs co-purify with a nascent cellulose chain

CesA is a processive GT that ‘holds on’ to the nascent cellulose polymer between elongation and translocation steps. Studies on secondary cell wall CesAs demonstrated that recombinantly expressed CesAs are catalytically active in the expression host. Accordingly, all experimentally determined plant CesA structures reveal the presence of a cellooligosaccharide inside the translocation channel. This is also observed for the primary cell wall soybean CesAs. Starting at the active site, the terminal glucosyl units of the nascent chains are positioned at the acceptor site next to the QxxRW motif’s Trp residue (Figure supplement 3b). This is consistent with the cellulose binding pose observed in bacterial CesA (Morgan et al., 2016). Generally, the first two glucosyl units are better resolved in the cryo EM maps, after which the densities become discontinuous. Constraint by the map qualities, we only modeled di- and hexasaccharides into the CesA3 and CesA6 structures, respectively.

### Interactions of homotrimers of different CesA isoforms

**Figure 3.**
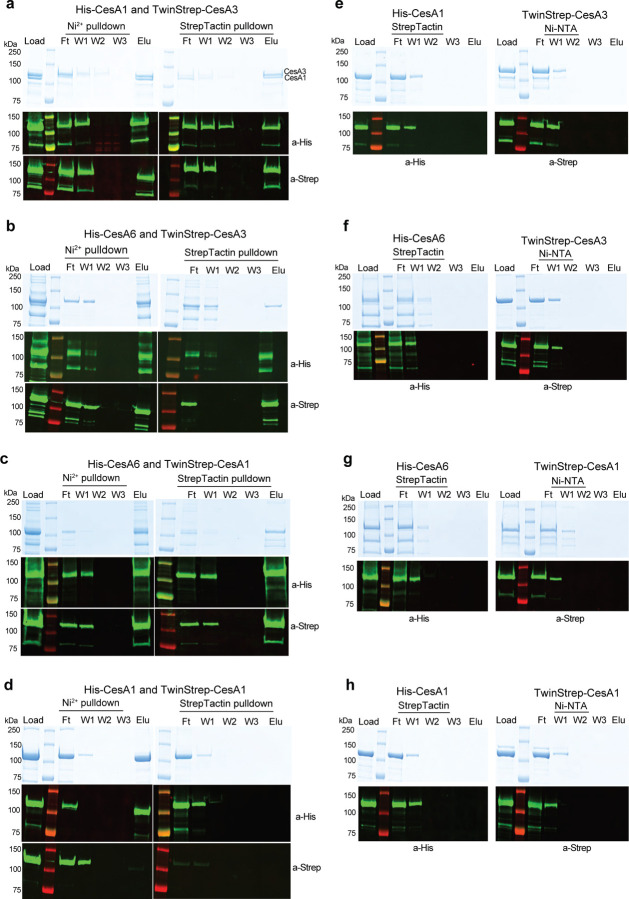
*In vitro* interactions between different CesA isoforms. Tandem pull-down experiments using Ni-NTA and Strep-Tactin resin. Experiments were performed with homotrimers of the indicated CesA isoforms tagged N-terminally either with His- or TwinStrep-tags. Material eluted from the Ni-NTA resin was loaded onto the Strep-Tactin beads. Top panels: Coomassie stained SDS-PAGE, bottom panels: Western blots using anti penta-His or anti-Strep primary antibodies. **(a-c)** Trimer-trimer interactions between CesA1 and CesA3, CesA6 and CesA3, and CesA6 and CesA1, respectively. **(d)** Differently tagged homotrimers of the same isoform do not interact. Tandem purification of a mixture of His- and TwinStrep-tagged CesA1. **(e-h)** Control binding of His-CesAs to Strep-Tactin beads and Strep-CesAs to Ni-NTA resin. F, W, E: Flow through, wash, and eluted fractions.

We next tested whether CesA homotrimers of different isoforms interact *in vitro*. To this end, we individually expressed and purified trimers of poly-His tagged CesA1 and CesA6 (His-CesA1 and His-CesA6) and TwinStrep-tagged CesA1 and CesA3 (Strep-CesA1 and Strep-CesA3) (Figure 3). Cross-isoform interactions were tested by tandem purifications over Ni-NTA and Strep-Tactin affinity matrices. His-CesA1 can be distinguished from Strep-CesA3 by Coomassie stained SDS-PAGE due to size differences, whereas all other species comigrate. Therefore, Western blotting together with Coomassie stained SDS-PAGE was performed to evaluate all co-purification results.

An equimolar mixture of His-CesA1 and Strep-CesA3 was incubated for 180 min on ice and sequentially purified using (1) Ni-NTA resin and (2) Strep-Tactin beads (Figure 3a). The Coomassie stained SDS-PAGE resolves both CesA species in the initial mixture, after elution from the Ni-NTA resin, as well as upon elution from the Strep-Tactin beads. The identity of the bands as His-CesA1 and Strep-CesA3 was confirmed by Western blotting. We observed no non-specific binding of His-CesA1 to Strep-Tactin beads or Strep-CesA3 to Ni-NTA resin (Figure 3e).

Similar experiments with combinations of Strep-CesA3 and His-CesA6 (Figure 3b) and Strep-CesA1 and His-CesA6 (Figure 3c) yielded comparable results, although the co-eluting species cannot be distinguished by Coomassie staining alone, due to comigration. None of the species showed detectable non-specific binding to the affinity resins in the absence of the corresponding tags, (Figure 3e–g).

As an additional control, we analyzed whether differently tagged homotrimers of the same isoform also interact with each other. To this end, a mixture of His-CesA1 and Strep-CesA1 was subjected to tandem affinity purification as described above (Figure 3d). However, we failed to detect any co-purification of Strep-CesA1 when applying Ni-NTA as the first affinity chromatography step. Similar results were obtained for Strep- and His-tagged combinations of homotrimers of CesA3 or CesA6 (Figure supplements 4a-d). Qualitatively, our interaction data are consistent with previously published co-immunoprecipitations of CesA isoforms (Gonneau et al., 2014; Timmers et al., 2009).

Similar tandem purification experiments were also performed with the monomeric CesA fractions obtained from size exclusion chromatography (Figure 1a–c). As observed for the homotrimeric complexes, monomeric His-CesA1 co-purifies with monomeric Strep-CesA3, demonstrating that trimeric assemblies are not necessary for the observed interactions (Figure supplements 4e and f).

### Homotrimers of different CesA isoforms assemble into clusters

**Figure 4.**
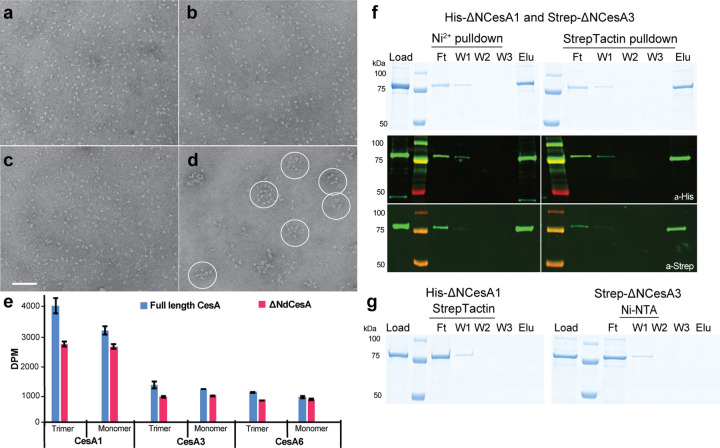
Clustering of CesA homotrimers. **(a-c)** Uranylformate stained EM images of homotrimers of purified GmCesA1 (A), GmCesA3 **(b)** and GmCesA6 **(c)**. The proteins were incubated overnight on ice prior to grid preparation. **(d)** The same for an equimolar mixture of all three CesA isoforms, incubated overnight, separated from individual trimers by size exclusion chromatography, and imaged by negative stain EM. Selected clusters are encircled. Scale bar: 100 nm. **(e-g)** Isoform interactions do not depend on the NTD. **(e)** Activity comparison of full-length and N-terminally truncated CesA isoforms. DPM: disintegrations per minute. **(f)** Tandem pull-down experiments as in Figure 3 but with N-terminally truncated homotrimers of CesA1 and 3. Top panel: SDS-PAGE, bottom panel: Western blots using anti-His and anti-Strep primary antibodies. **(g)** Control binding of His-ΔNCesA1 to Strep-Tactin beads and Strep-ΔNCesA3 to Ni-NTA resin. F, W, E: Flow through, wash, and eluted fractions.

Negative stain electron microscopy of the individual CesA homotrimers revealed monodispersed particle distributions for all three CesA isoforms (Figure 4 a–c). The size and shape of the particles is consistent with CesA homotrimers as observed by cryo EM and remained unchanged over a course of 4 days when the samples were stored on ice. In addition, all three homotrimeric isoforms were combined and subjected to size exclusion chromatography after incubation overnight on ice. Negative stain EM analysis of high molecular weight fractions eluting after the void volume revealed CesA clusters of varying stoichiometries (Figure 4d). The clusters likely arise from trimer interactions in different orientations and range in diameter from about 50 to 100 nm. No clustering was observed for the individual CesA isoforms alone, even after prolonged incubation (4 days) on ice.

### CesA isoform interaction does not require the N-terminal domain

The biological function of CesA’s N-terminal domain (NTD) and CSR are unknown. While the CSR is located at the corners of the CesA trimer (Figure 2e), the NTD extends into the cytosol as a stalk or adopts multiple conformations that are unresolved in cryo EM reconstructions (Figure supplement 2). The NTD’s RING-like domain has been shown to form dimers and trimers *in vitro* (Kurek et al., 2002; Purushotham et al., 2020), raising the possibility of forming inter-trimer complexes accounting for the observed isoform interactions.

To test this hypothesis, N-terminally truncated constructs of CesA1 and CesA3 were expressed and purified as described for the full-length variants. The constructs lack the first 259 (CesA1) and 242 (CesA3) residues yet purify as trimers (besides monomers) and exhibit *in vitro* catalytic activity similar to the full-length constructs (Figure 4e). A tandem affinity purification of a mixture of the truncated His-CesA1 and Strep-CesA3 isoforms demonstrates their interaction *in vitro*, as observed for the full-length enzymes (Figure 4f and g and Figure 3a). This suggests that the NTD is not required for inter-isoform interactions of CesAs *in vitro*.

### Synergistic cellulose biosynthesis

**Figure 5.**
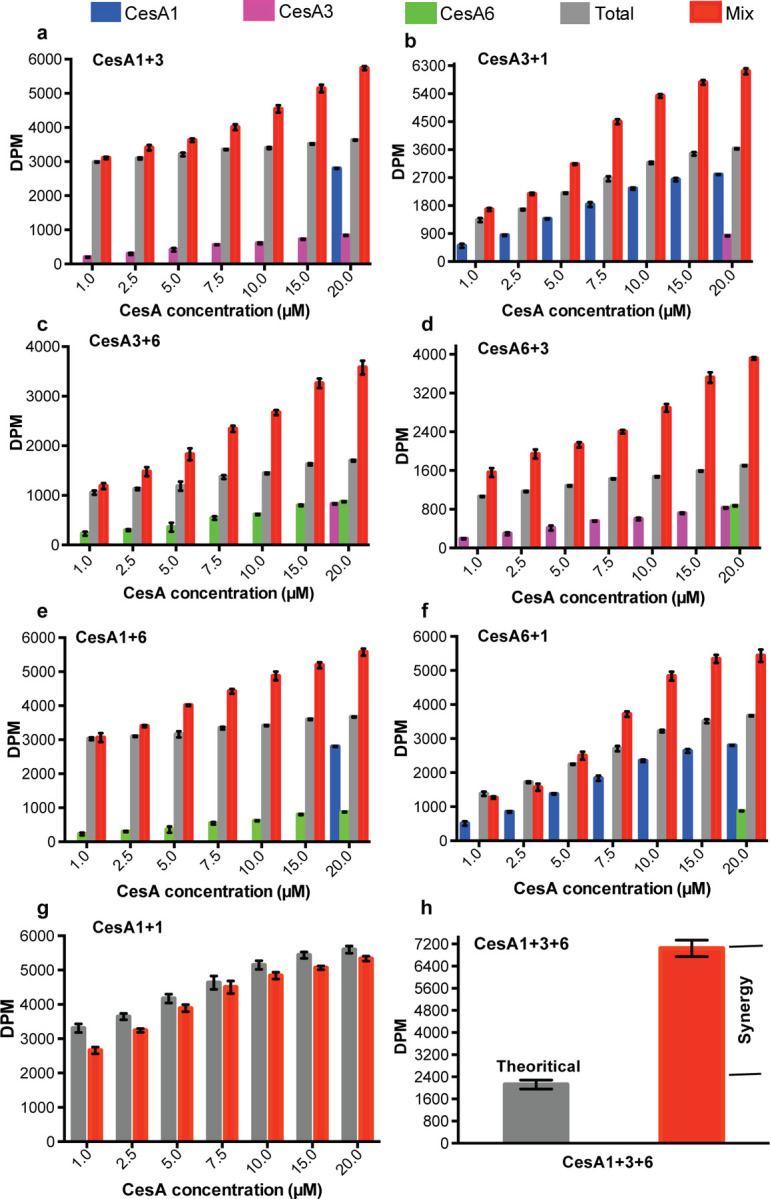
Synergistic catalytic activity. Cellulose biosynthesis from mixtures of CesA isoforms. The formation of ^3^H-labeled cellulose was quantified by scintillation counting for reaction mixtures containing one CesA isoform at 20 µM concentration and the other isoform at the indicated increasing concentrations. Blue, magenta and green columns represent activities measured for the individual single isoforms alone. Gray columns represent the calculated theoretical activities for the isoform mixtures by adding the individually determined activities. Red columns represent the experimentally determined activities for the isoform mixtures. **(a and b)** CesA1_20 μM_ + CesA3_1– 20 μM_ and CesA3_20 μM_ + CesA1_1– 20 μM_; **(c and d)** CesA3_20 μM_ + CesA6_1– 20 μM_ and CesA6_20 μM_ + CesA3_1– 20 μM_; and (**e and f**) CesA1_20 μM_ + CesA6_1– 20 μM_ and CesA6_20 μM_ + CesA1_1–20 μM_, respectively. **(g)** The same as for panel **(a)** but titrating the same CesA isoform (CesA1_20 μM_ + CesA1_1– 20 μM_). **(h)** The same as for panels a-f but for a combination of all three CesA isoforms. Individual and combined activities were determined at a concentration of 6.6 µM for each CesA isoform. DPM, disintegrations per minute.

We asked whether cross-isoform interactions of CesAs modulate their *in vitro* catalytic activities. To this end, *in vitro* cellulose biosynthesis was quantified radiometrically from reactions containing one CesA isoform at a constant concentration (20 µM) and increasing concentrations of the other (1–20 µM). As a reference, cellulose biosynthetic activities were also determined for each isoform alone at the concentrations used in the combined assays. As shown in Figure 5A–F, for all isoform combinations, the measured activities exceed the theoretical activities (calculated by adding the individually measured activities) at least one to two-fold, depending on the isoform combination. This suggests synergistic cellulose biosynthesis in the presence of two different CesA isoform trimers. Performing the titration experiment with samples of the same isoform does not exhibit synergy, consistent with the lack of interactions of trimers of the same isoform (Figure 5g and 3d - figure supplement 5).

Additionally, comparing the measured and additive activities obtained after combining all three CesA isoforms (at 6.6 µM each) reveals an experimental activity about 3-fold above the theoretical value. This activity level may arise from different dimeric arrangements of CesA trimers (1+3, 1+6, and 3+6) and/or the formation of trimeric complexes of different isoform trimers (1+3+6) (Figure 5h).

Activity measurements with the individual CesA isoforms suggest about 4-fold higher catalytic activity of CesA1 compared to CesA3 and CesA6 (Figure 1d). To test whether the observed synergistic effects are due to altered catalytic activity of only one isoform or both, one CesA isoform was inactivated after purification by incubation with the oxidant sodium tetrathionate (Tie et al., 2004). While the inactivated CesAs exhibit activity levels comparable to EDTA-treated negative controls, they remain trimeric and interact with other isoforms as observed for the unmodified versions (Figure supplement 6). Inactivation could be due to modification of a conserved cysteine residue in CesA’s catalytic pocket (such as Cys558 in CesA6).

Performing the above-described activity assays with pairs of inactive and active CesA trimers demonstrates that all three isoforms exhibit increased catalytic activity when combined with an inactive trimer of a different isoform (Figure supplement 7). This suggests that intertrimer interactions impact the catalytic activity of all isoforms, perhaps by altering the accessibility of the catalytic pocket (discussed below).

## Discussion

A hallmark of plant cellulose biosynthesis is the formation and arrangement of cellulose microfibrils in the cell wall (Zhang et al., 2021b). Prevailing models postulate the requirement of CesA supramolecular complexes (CSC) of at least three different isoforms (Newman et al., 2013; Turner and Kumar, 2018) for proper cell wall biogenesis of primary and secondary cell walls. However, experimental evidence directly pinpointing isoform locations within a CSC or in its repeating unit is lacking.

Our attempts to isolate heterotrimeric complexes of co-expressed GmCesA1, GmCesA3 and GmCesA6 failed, despite sufficient expression levels of all isoforms. Thus, we shifted our attention to characterizing the isoforms individually. All three isoforms can be purified as catalytically active homotrimeric species. Trimerization involves the same structural motifs identified in CesA8 and CesA7, primarily the cytosolic PCR domain. Because the PCR is highly conserved across the three isoforms, the apparent failure of heterotrimer formation may be due to subtle differences in sequence and shape complementarity of the isoforms.

Differently arranged TM helices in primary cell wall CesAs compared to CesA8 and CesA7 create a large lateral window in the cellulose secretion channel. The window opens towards the trimer’s 3-fold symmetry axis. While the biological function of this window is unclear, it could enable the lateral release of the glucan chains towards the center of the complex, thereby facilitating the alignment of the nascent glucan chains into a protofibril.

**Figure 6.**
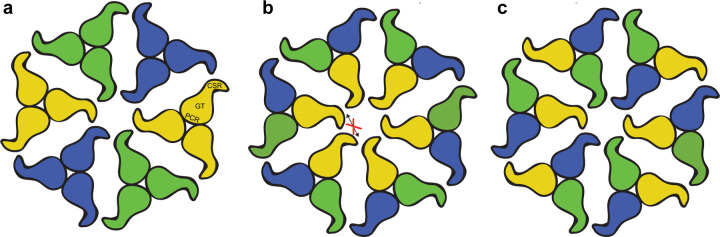
CSC models consisting of different CesA isoforms. **(a)** Association of homotrimers of three different CesA isoforms. Isoforms are indicated by different colors. The shapes represent the cytosolic CesA domains. **(b and c)** CSC assembly from heterotrimeric CesA complexes. Neither heterotrimer formation nor interactions between the same isoforms have been observed *in vitro,* rendering the models less likely. PCR: Plant conserved region, GT: Glycosyltransferase, CSR: Class specific region.

Our *in vitro* CesA interaction studies replicate previous *in vivo* co-immunoprecipitation results on primary and secondary cell wall CesAs (Gonneau et al., 2014; Timmers et al., 2009). The robust interaction of different CesA isoform trimers supports their physiological significance. In a detergent solubilized state, the individual CesA trimers are not confined to the same plane, as would be the case in a biological membrane. Thus, the *in vitro* observed interactions lead to clustering of the trimers, instead of an ordered array into symmetric particles. Isoform interactions do not require the N-terminal domain containing the dimerization-prone RING-like domain. Therefore, we propose the CSR domain as the primary mediator of trimer-trimer interactions (Figure 6), in agreement with earlier interpretations (Sethaphong et al., 2013; Singh et al., 2020). This model is supported by the CSR’s hook-like extension from the CesA trimer. We assume distinct binding sites for two ‘non-like’ isoforms within a CSR. A dimeric complex of, for example CesA1 and CesA3, would then only be able to interact with CesA6, thereby explaining the functional importance of three CesA isoforms. This model can be modified by postulating multiple binding sites for the same ‘non-like; isoform or even a ‘like’ isoform to account for possible CSC configurations of two or one isoform. Because trimers of the same isoform do not interact in vitro, CSC models relying on interactions between the same CesA isoforms across the repeat units are unlikely (Figure 6).

Because the CSR is a structurally flexible domain (Scavuzzo-Duggan et al., 2018) that likely occupies a large volume at the periphery of the catalytic domain, it is possible that it impacts nucleotide binding to or exchange at the active site. The CSR could be repositioned upon interaction with another isoform, which in turn could increase catalytic activity and account for the observed synergistic effects.

Lastly, the biological functions of the different CesA isoforms are currently unknown. Requiring different isoforms to form a functional CSC could provide regulatory control over the cellulosic material that is deposited in the cell wall. It is conceivable that CesA trimers function alongside fully assembled CSCs in the plasma membrane, thereby producing micro- and protofibrils (from CSCs and trimers, respectively) that may interact. Accordingly, controlling the ability of the CesAs to assemble into CSCs by regulating the isoform composition in the plasma membrane may allow tailoring the fibril-to-protofibril ratio and thereby wall properties. Addressing these questions will require detailed studies of the oligomerization and distribution of cellulose depositing CesA complexes in the plasma membrane.

## Materials and methods

### GmCesA protein expression and purification

#### Cloning

The primary cell wall CesA1, 3 and 6 genes from soybean were synthesized (Gene Universal) with an N-terminal 12x His-tag coding sequence and cloned into *Not*I and *Hind*III restriction sites in the pACEBac1vector. The N-terminally TwinStrep-tagged CesA1 and CesA3 constructs were generated by QuikChange mutagenesis from the pACEBac1–12xHisCesA vectors. The N-terminally deleted GmCesA constructs (∆NCesAs) were generated by PIPE cloning from the full-length constructs, resulting in plasmids pACEBac1-∆NCesA1R260, pACEBac1-∆NCesA3V243, and pACEBac1-∆NCesA6M248.

#### Virus generation

An aliquot of 3 μL of 100 ng/μL pACEBac1-CesA plasmid was used for transformation into 50 μL chemical-competent DH10MultiBacTurbo *E. coli* cells. Bacmids were isolated from white colonies on a Bluo-Gal agar plate and transfected into *Spodoptera frugiperda* 9 (SF9) cells. P0, P1 and P2 baculovirus was generated according to the Joint Centre for innovative membrane protein technologies (JCIMPT) protocol.

#### Protein expression and purification

For GmCesAs expression, SF9 insect cells were infected with a baculovirus at a density of 3 M cells per mL and grown at 27 °C for 48–72 h in an orbital shaker. Cells were then harvested by centrifugation at 5,000xg for 10 min at 4 °C. Cell pellets were resuspended in buffer A (20 mM Tris-HCl, pH 7.5, 100 mM NaCl, 5 mM sodium phosphate, 5 mM sodium citrate, 1 mM TCEP, 1 μM leupeptin, 1 μM pepstatin, 0.5 μM aprotinin, 1 mM AEBSF, 100 μM bestatin, 10 μM E-64 and 5 mM Benzamidine HCl) containing 1% Lauryl Maltose Neopentyl Glycol (LMNG, Anatrace) and 0.2% cholesteryl hemisuccinate (CHS, Anatrace) and lysed in a glass dounce homogenizer  (~ 30 strokes). The lysate was solubilized at 4°C for 1 h in a rotator shaker. After separation of insoluble material by centrifugation at 204,709 x g for 30 min, 5 mL of Ni-NTA resin (HisPur Ni-NTA Resin, Thermo scientific) was added to the supernatant and incubated for 1 h at 4 °C in a rotator shaker. The resin was then sequentially washed, wash 1 and wash 2, with 10 column volumes each of buffer A containing 40 mM imidazole, 0.02% glyco-diosgenin (GDN, Anatrace), followed by 10 column volumes of buffer A containing 1 M NaCl and 0.02% GDN (wash 3). The final wash step (wash 4) was with buffer A containing 0.02% GDN and 60 mM imidazole. The GmCesAs were eluted with five column volumes of buffer A containing 0.02% GDN and 400 mM imidazole.

The TwinStrep tagged GmCesAs was affinity purified by incubating the membrane extract with Strep-Tactin sepharose at 4 °C for 1 h in a rotator shaker. After batch binding, the resin was packed into a gravity flow column and washed as mentioned above for Ni-NTA affinity column except the buffers lacked imidazole. The TwinStrep-tagged CesA was eluted using 10 column volumes of buffer A containing 0.02% GDN and 5 mM desthiobiotin.

His- and TwinStrep-tagged GmCesAs were further purified by size-exclusion chromatography (SEC) using Superose 6 Increase 10/300 GL column (Cytiva) equilibrated in buffer A containing 0.02% GDN without any protease inhibitors. The purified GmCesA trimers and monomers were immediately used for activity assays and pulldown experiments or flash-frozen in liquid nitrogen and stored at −80°C.

#### CesA-CesA pull-down assays

After SEC, either the trimeric or monomeric fractions of His-CesA and TwinStrep-CesA were pooled and used for tandem affinity chromatography using Ni-NTA and Strep-Tactin sepharose beads. His-CesA1 and TwinStrep-CesA3 trimers were mixed at 150 μg/mL concentration and incubated at 4 °C for 3 h. The mixture was first loaded onto 200 μL bed volume Ni-NTA beads for 1 h at 4 °C in the presence of 20 mM imidazole. After collecting the flowthrough, the beads were washed with 10 column volumes of buffer A containing 0.02% GDN and 20 mM imidazole for 3 washes followed by elution in 5 column volumes of buffer A containing 0.02% GDN and 400 mM imidazole.

The Ni-NTA eluted material was next bound to 200 μL Strep-Tactin sepharose beads for 1 h at 4 °C in a rotator shaker. After batch binding, the beads were packed into a gravity flow column. The flow-through was collected and the column was subsequently washed 3-times with 10 column volumes of buffer A containing 0.02% GDN. Bound proteins were eluted with buffer A containing 0.02% GDN and 5 mM desthiobiotin. All fractions were analyzed by SDS-polyacrylamide gel electrophoresis (SDS-PAGE) and Western blotting using anti-His (Qiagen) and anti-strep (MilliporeSigma) primary antibodies and an IRDye800-coupled anti-mouse secondary antibody (Rockland) for detection.

The same protocol was used to study the interactions of CesA1 and CesA6 or CesA3 and CesA6 using Strep-tagged CesA1 and His-tagged CesA6, Strep-tagged CesA3 and His-tagged CesA6, respectively. Control binding experiments were performed by loading the His-tagged CesA species onto Strep-Tactin sepharose beads and the Strep-tagged CesAs onto the Ni-NTA beads. In each case, the beads were washed as described above.

#### Inactivation of CesA

CesA was inactivated by treating CesA trimers with 10 mM sodium tetrathionate overnight at room temperature (24 °C). The treated sample was purified over a size exclusion column to remove the excess sodium tetrathionate. Binding experiments with inactivated and untreated CesAs were performed using Ni-NTA beads as described above.

#### GmCesA activity assay

Freshly purified or aliquots of flash-frozen enzyme thawed on ice were used for activity assays. In general, activity assays were performed as described earlier (Carpita, 2011; Purushotham et al., 2020; Wilson et al., 2021). Radiometric quantification of *in vitro* synthesized cellulose was performed by combining 5 μM GmCesA, 5 mM UDP-glucose (UDP-Glc), and 0.25 μCi UDP-[^3^H]-Glc in buffer containing 20 mM Tris-HCl, pH 7.5, 100 mM NaCl, 20 mM MgCl_2_, 5 mM sodium phosphate, 5 mM sodium citrate,1 mM TCEP. The reaction mixtures were incubated for 45 min at 37°C. After incubation, the entire reaction mixture was spotted on Whatman Grade 3MM chromatography paper. Free substrate was removed by descending paper chromatography in 60% ethanol. The radioactivity retained at the origin was quantified by scintillation counting.

The pH optima of GmCesAs were determined by incubating the CesAs in MMT buffer, consisting of DL-malic acid, MES and Tris base in the molar ratios 1:2:2-DL-malic acid:MES:Tris base. The desired pH was adjusted with NaOH or HCl. Activity assay was performed as mentioned above in triplicate from two biological replicates.

Cellulase digestions were performed by adding 5 U of endo-β−1,4-glucanase (*Trichoderma longibrachiatum*; Megazyme: E-CELTR) directly to the reaction mixture. Post *in vitro* synthesis reaction, cellulase treatment was performed for 3 h at 37°C. Cellulose quantification by scintillation counting was performed as mentioned above to quantify the product.

Steady-state kinetic analyses were performed in triplicate using the UDP-Glo Glycosyltransferase Assay kit (Promega) to monitor the released UDP according to the manufacturer’s instructions. For measuring enzyme kinetics, the reaction mixtures containing 5 μM GmCesA, 0.005–5 mM UDP-Glc were added to 20 mM Tris-HCl buffer (pH 7.5), 100 mM NaCl, 5 mM sodium phosphate, 5 mM sodium citrate, 20 mM MgCl_2_ and incubated in a final volume of 25 μl for 45 min at 37°C. The reaction mixture was mixed with an equal amount of UDP-Glo reagent in a 96-well Nunclon Delta-Treated flat-bottom microplate (Thermo Scientific) and incubated for 1 h at room temperature before measuring luminescence using a GloMax Explorer plate reader (Promega). A standard curve was used for quantification of the UDP produced. Kinetic values were obtained using the nonlinear regression function in GraphPad Prism.

#### GmCesA UDP inhibition assays

UDP inhibition was analyzed using radiometric quantification of *in vitro* synthesized cellulose, as described above by titrating 0.01–7.5 mM UDP in the reaction. Substrate concentrations for the individual reactions matched the estimated Km values: CesA1: 1.4 mM; CesA3: 0.5 mM; CesA6: 2.3 mM. Inhibition constants (Ki) for each CesA were obtained by analyzing the data using GraphPad Prism software.

#### Synergistic cellulose biosynthesis

Activity synergism between different CesA isoform trimers were studied by mixing two CesA isoforms, one at a constant concentration of 20 μM and one at increasing concentrations from 1–20 μM. The activities of the individual isoforms at the respective concentrations were also measured. *In vitro* synthesized cellulose was quantified by scintillation counting, as described above.

#### EM grid preparation and data collection

After size exclusion chromatography, the freshly purified protein fractions were pooled. The protein quality was monitored by negative stain EM. The proteins were diluted to 0.01 mg/mL and 4 μL was applied to a glow discharged Formvar/Carbon grid (Electron Microscopy Sciences) for 30 s, followed by 2x washes with 4 μL H_2_O. The grid was negatively stained with 4 μl 0.75% Uranyl Formate (UF) in H_2_O for 30 s. Excess UF was removed by blotting with filter paper and the grid was air dried. Images were taken on a Tecnai F20 at Macromolecular Electron Microscopy Core (MEMC) facility at the University of Virginia.

For cryo grid preparation, the protein samples were concentrated to ~3 mg/mL (based on UV absorbance). Samples of 2.5 µL were applied to a C-flat 300 mesh 1.2/1.3 copper grids (Electron Microscopy Sciences), glow-discharged in the presence of amylamine at 25 mA for 45 s, and blotted with a Vitrobot Mark IV (FEI, Thermo Fisher Scientific) with force 4 for 6 s at 4°C, 100% humidity, and frozen in liquid ethane.

Cryo EM data were collected at the MEMC at the University of Virginia on a Titan Krios microscope operated at 300 keV and equipped with a Gatan K3 direct electron detector positioned post a Gatan Quantum energy filter. Total ~6000 movies were collected from 2 grids in counting mode at a magnification of 81K, pixel size of 1.08 Å, and defocus range from −2.2 to −1.2 µm with step size of 0.2 µm. The total dose was 50 e^−^/Å^2^. Movies with 40 frames were collected at 5.17 s/movie rate.

#### Cryo EM data processing

Cryo EM data processing was done in cryoSPARC v4 (Punjani et al., 2017). The general workflow is described in *SI Appendix*, Fig. S2. Based on improved map qualities, all volumes were generated imposing 3-fold symmetry (C3). Maps generated without symmetry assignment (C1) were used to visualize the unidentified ligand coordinated by the PCR domains at the particle’s symmetry axis. Initial protein models were generated in AlphaFold and manually adjusted in Chimera and Coot (Emsley and Cowtan, 2004; Pettersen et al., 2004). Models were refined in Phenix.refine (Afonine et al., 2012). The following regions were omitted from the constructs due to weak or missing map density: CesA1:1–260 (NTD), 654–717 (CSR), 954–978 (gating loop), 1064–1078 (C term); CesA3: 1–251 (NTD), 648–712 (CSR), 946–974 (gating loop), 1032–1079 (C term and TM 7); and CesA6: 1–248 (NTD), 646–712 (CSR), 952–971 (gating loop), 1058–1078 (C term). Structural representations were generated in ChimeraX or Pymol (Pettersen et al., 2021; Pymol).

## Data Availability

PDB coordinates and cryo EM maps have been deposited at the PDB with accessing codes 8VHZ, 8VHT, and 8VIO.
