# Supplementary material for: Structure, function and assembly of soybean primary cell wall cellulose synthases
Source: bioRxiv. 2025 Jan 14:2024.02.13.580128. Originally published 2024 Feb 15. Preprint. [Version 2] doi: 10.1101/2024.02.13.580128 (PMC10888898; doi:10.1101/2024.02.13.580128)
Supplement: Supplement 1 [file NIHPP2024.02.13.580128v2-supplement-1.pdf]

bioRxiv preprint doi: https://doi.org/10.1101/2024.02.13.580128; this version posted January 14, 2025. The copyright holder for this preprint (which was not certified by peer review) is the author/funder, who has granted bioRxiv a license to display the preprint in perpetuity. It is made available under aCC-BY 4.0 International license.

Supplemental Figure 1-8

Supplemental Table 1

bioRxiv preprint doi: https://doi.org/10.1101/2024.02.13.580128; this version posted January 14, 2025. The copyright holder for this preprint (which was not certified by peer review) is the author/funder, who has granted bioRxiv a license to display the preprint in perpetuity. It is made available under aCC-BY 4.0 International license.

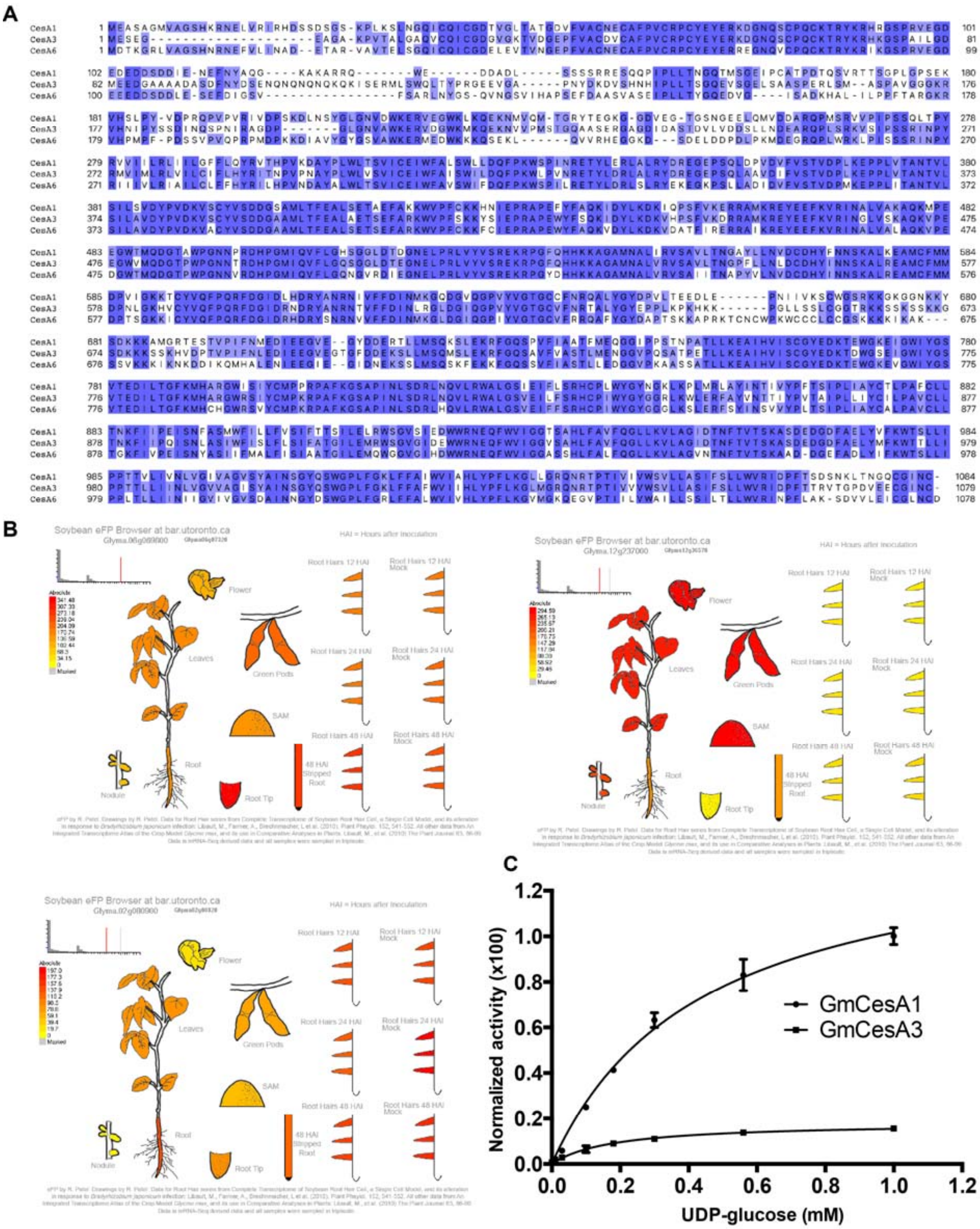

Figure 1. Sequence alignment of soybean CesA1, CesA3, and CesA6 and substrate turnover kinetics. A) Sequences were aligned in Clustal Omega (Larkin *et al.*, 2007) and visualized in Jalview (Waterhouse et al., 2009) showing sequence identity from dark blue (high) to white

bioRxiv preprint doi: https://doi.org/10.1101/2024.02.13.580128; this version posted January 14, 2025. The copyright holder for this preprint (which was not certified by peer review) is the author/funder, who has granted bioRxiv a license to display the preprint in perpetuity. It is made available under aCC-BY 4.0 International license.

(low). **B**) Gene expression profiles of soybean GmCesA1: Glyma.06G069600, GmCesA3: Glyma.12G237000, and GmCesA6: Glyma.02G080900 from the soybean eFP Browser (https://bar.utoronto.ca/efpsoybean/cgi-bin/efpWeb.cgi). **C**) In vitro Michaelis Menten kinetic analysis of CesA1 and CesA3 by titrating UDP-Glc and quantifying the generated UDP using an UDP-Glo assay kit. The data is normalized to the highest activity of GmCesA1. The activity of GmCesA6 was too weak to be analyzed by this method. Error bars represent the standard deviations from the means of three technical replicas.

bioRxiv preprint doi: https://doi.org/10.1101/2024.02.13.580128; this version posted January 14, 2025. The copyright holder for this preprint (which was not certified by peer review) is the author/funder, who has granted bioRxiv a license to display the preprint in perpetuity. It is made available under aCC-BY 4.0 International license.

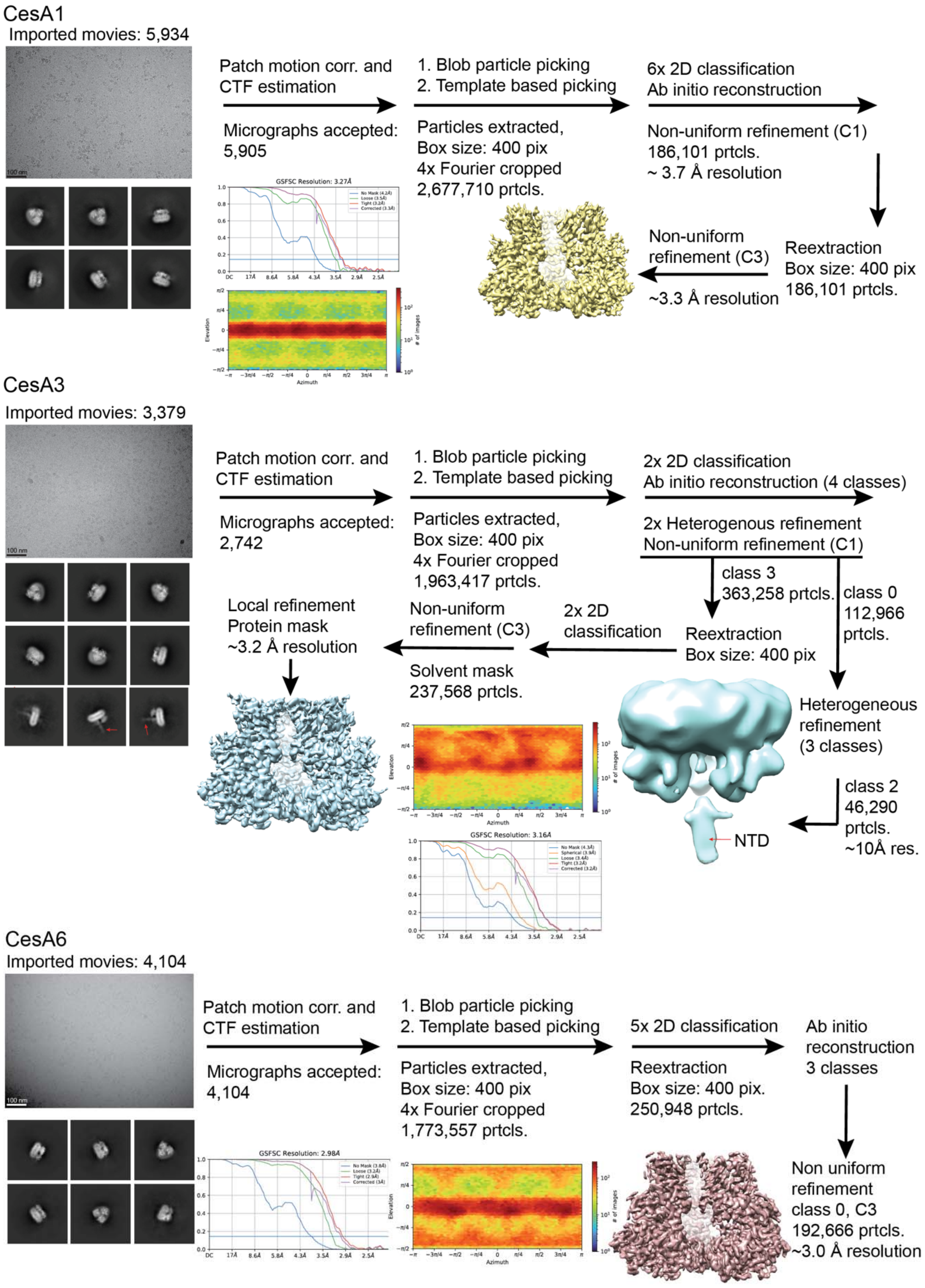

Figure 2. Cryo-EM data processing workflows.

All steps were performed in CryoSparc v4 (Punjani *et al.*, 2017).

bioRxiv preprint doi: https://doi.org/10.1101/2024.02.13.580128; this version posted January 14, 2025. The copyright holder for this preprint (which was not certified by peer review) is the author/funder, who has granted bioRxiv a license to display the preprint in perpetuity. It is made available under aCC-BY 4.0 International license.

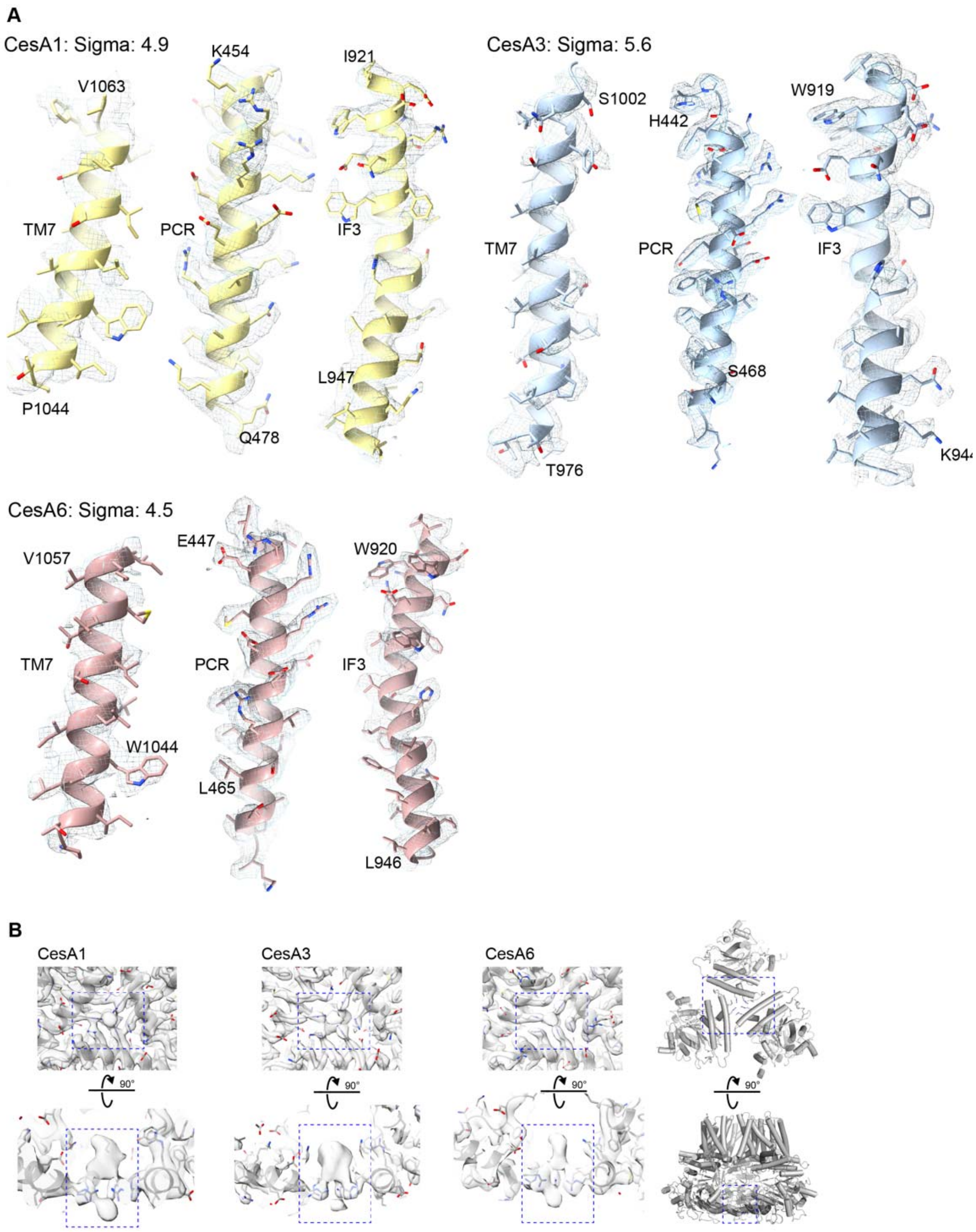

Figure 3. Cryo-EM map quality examples.

A) Selected helical segments of CesA1, CesA3, and CesA6. TM helix 7 was excluded from the GmCesA3 model due to limiting map quality. **B)** Close-up views of the unidentified ligands coordinated by the PCR domains of the homotrimers.

bioRxiv preprint doi: https://doi.org/10.1101/2024.02.13.580128; this version posted January 14, 2025. The copyright holder for this preprint (which was not certified by peer review) is the author/funder, who has granted bioRxiv a license to display the preprint in perpetuity. It is made available under aCC-BY 4.0 International license.

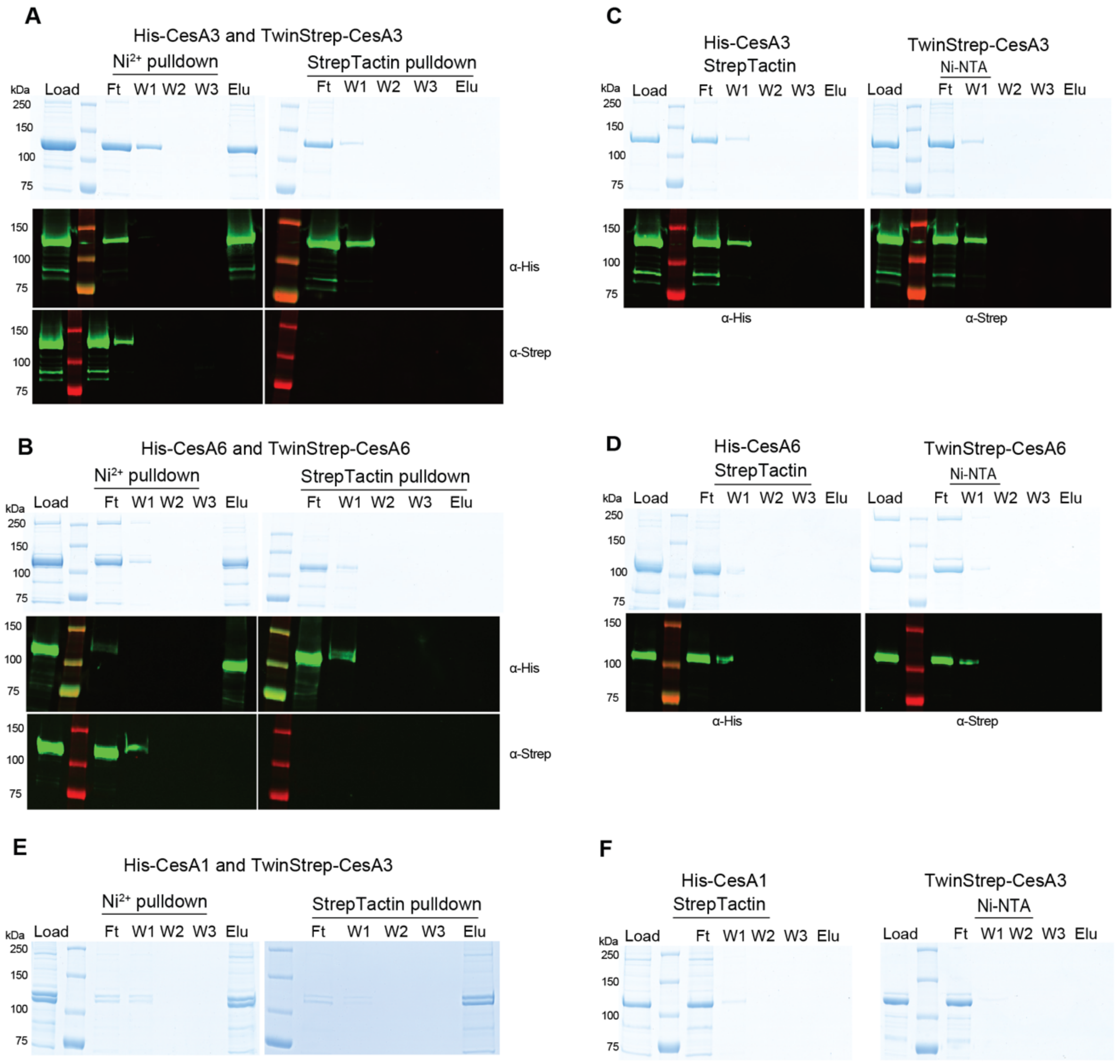

Figure 4. Interactions of CesA homotrimers of the same isoforms.

A-D) Tandem purifications of combinations of His- and Strep-tagged CesA3 or CesA6 (A and B) together with the non-specific binding controls (C and D). (E and F) Interactions of monomers of different CesA isoforms. **E)** Monomeric versions of His-tagged CesA1 and Strep-tagged CesA3 were subjected to tandem purification starting either with Ni-NTA resin or Strep-Tactin beads. **F)** Controls to account for non-specific interactions.

bioRxiv preprint doi: https://doi.org/10.1101/2024.02.13.580128; this version posted January 14, 2025. The copyright holder for this preprint (which was not certified by peer review) is the author/funder, who has granted bioRxiv a license to display the preprint in perpetuity. It is made available under aCC-BY 4.0 International license.

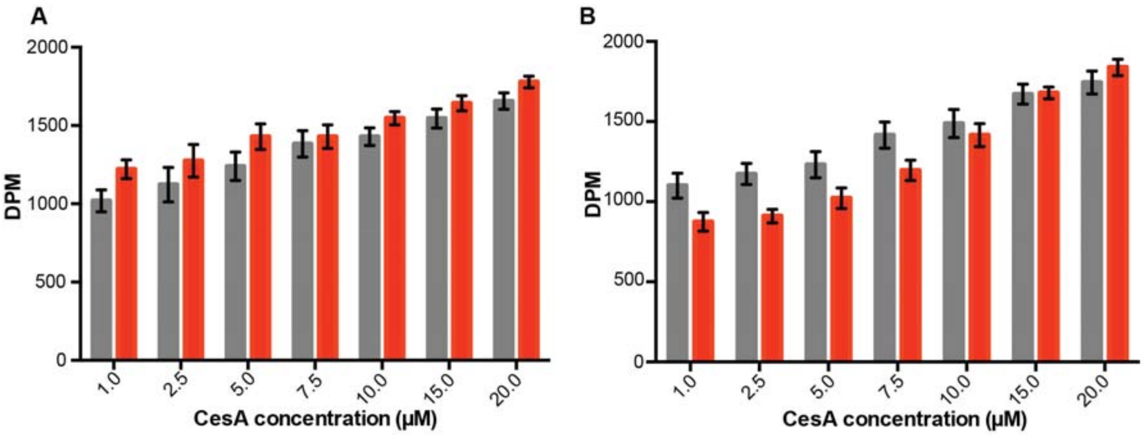

Figure 5. Control synergistic activity assays by titrating the same CesA isoforms.
A) CesA3$_{20\ \mu M}$ + CesA3$_{1-\ 20\ \mu M}$, B**)** CesA6$_{20\ \mu M}$ + CesA6$_{1-\ 20\ \mu M}$. Gray columns indicate calculated theoretical activities from individual measurements, red columns are experimentally determined activities. DPM: Disintegrations per minute.

bioRxiv preprint doi: https://doi.org/10.1101/2024.02.13.580128; this version posted January 14, 2025. The copyright holder for this preprint (which was not certified by peer review) is the author/funder, who has granted bioRxiv a license to display the preprint in perpetuity. It is made available under aCC-BY 4.0 International license.

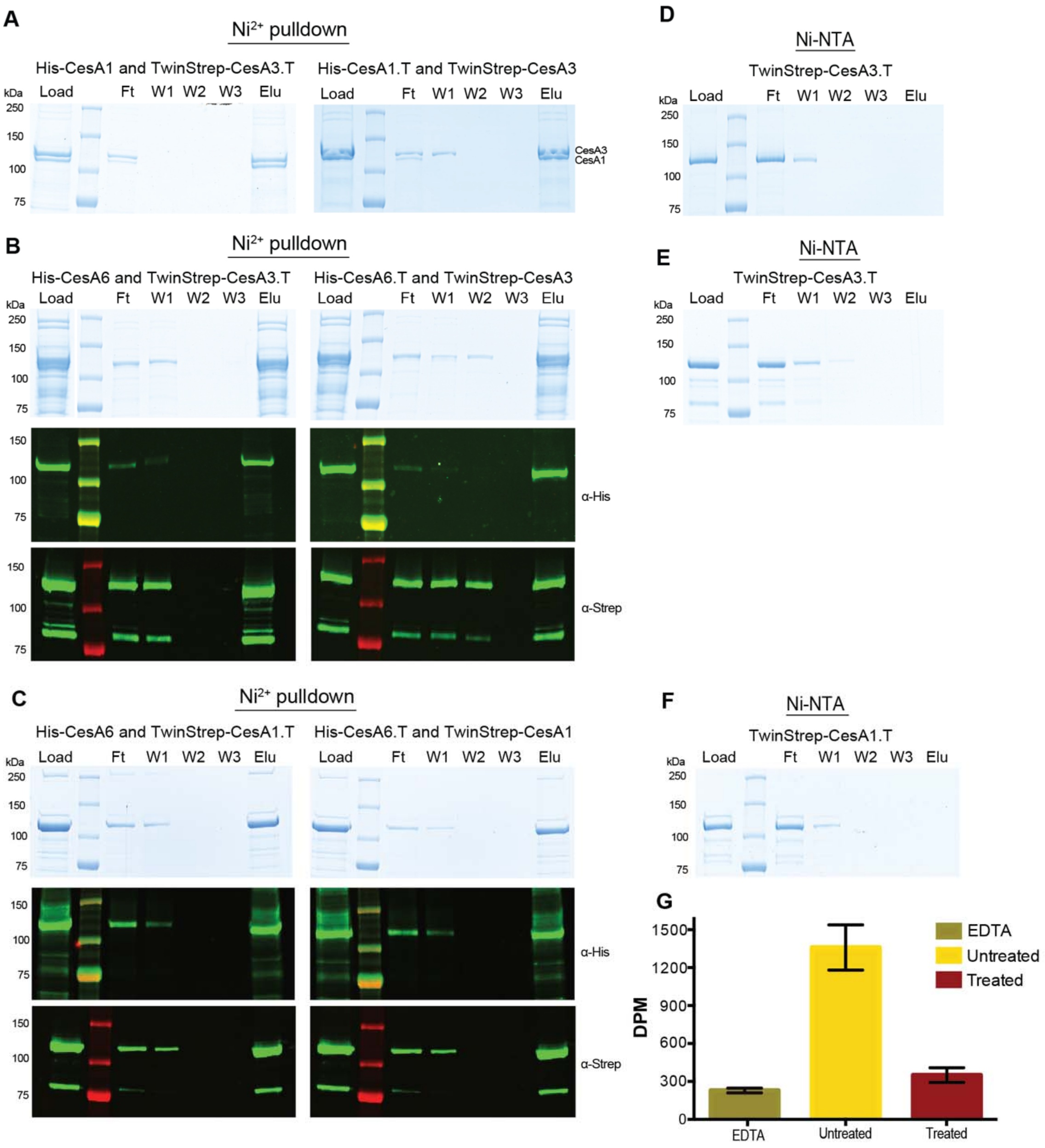

Figure 6. Tetrathionate-inactivated CesAs interact with other CesA isoforms.

A-C) Co-purification of catalytically active CesA homotrimers with tetrathionate inactivated CesA homotrimers (indicated by a .T extension). The indicated combinations of CesAs were purified over Ni-NTA beads. Results were analyzed by Coomassie stained SDS-PAGE (top panels) and Western blotting (bottom panels). **D-F)** Control binding experiments of Strep-tagged CesAs and Ni-NTA resin. **G)** Catalytic activity of tetrathionate treated GmCesA3.

bioRxiv preprint doi: https://doi.org/10.1101/2024.02.13.580128; this version posted January 14, 2025. The copyright holder for this preprint (which was not certified by peer review) is the author/funder, who has granted bioRxiv a license to display the preprint in perpetuity. It is made available under aCC-BY 4.0 International license.

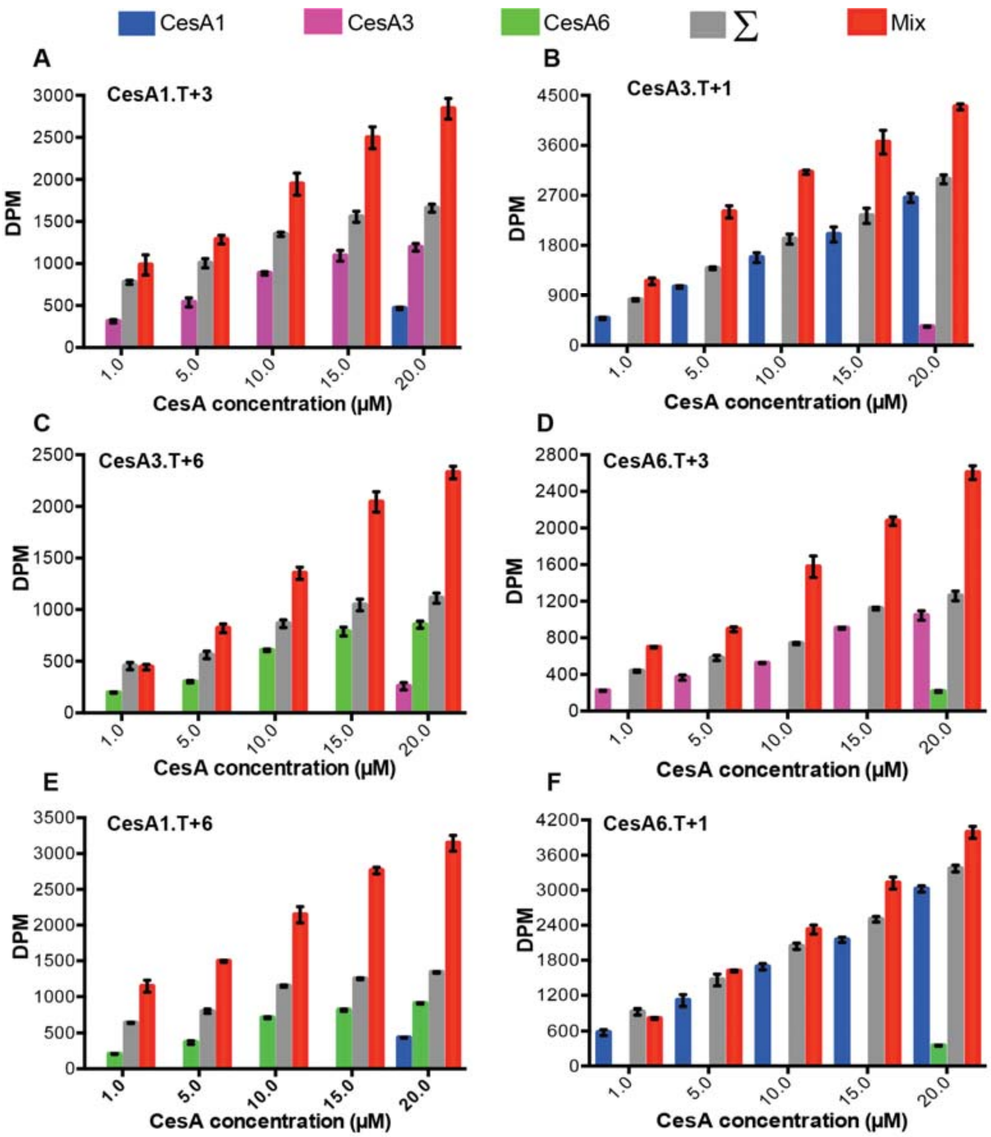

Figure 7. Synergistic cellulose biosynthesis with tetrathionate inactivated CesA trimers.
A-F) Inactivated CesA trimers (indicated by a '.T' extension) at a constant concentration were incubated with increasing concentrations of wild type CesAs of a different isoform. Background activities of the inactivated CesAs range from ~200-400 DPM. Calculated total theoretical activities are shown as gray columns, experimentally determined activities are shown as red columns. DPM: disintegrations per minute.

bioRxiv preprint doi: https://doi.org/10.1101/2024.02.13.580128; this version posted January 14, 2025. The copyright holder for this preprint (which was not certified by peer review) is the author/funder, who has granted bioRxiv a license to display the preprint in perpetuity. It is made available under aCC-BY 4.0 International license.

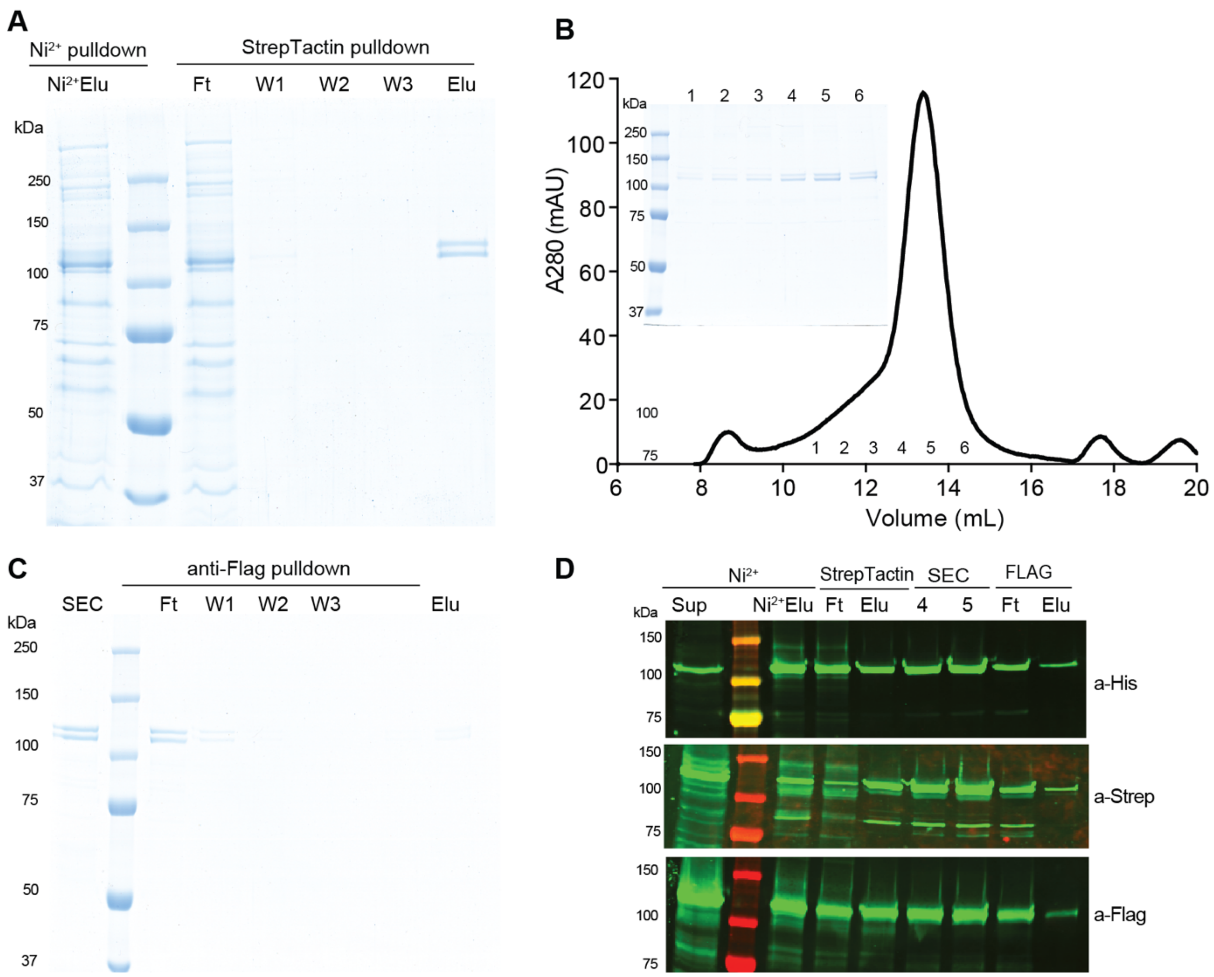

Figure 8. Attempt to purify hetero-oligomeric CesA oligomers. GmCesA1, GmCesA3, and GmCesA6 were co-expressed in Sf9 cells and purified by sequential affinity chromatography. **A**) Material eluted (Elu) from a Ni-NTA column was purified over Strep-Taction affinity resin. **B**) The Elu fraction from the Strep-Tactin resin was loaded onto a Superose-6 size exclusion chromatography column (SEC). The inset shows a Coomassie stained SDS-PAGE of the indicated fractions. **C**) The peak fraction from (B) was loaded onto an anti-Flag affinity matrix, washed (W), and eluted. **D**) Western blot analysis of eluting (Elu), flow through (Ft), and SEC (4, 5) fractions from all steps shows the presence of all CesA species, alongside significant loss in all Ft fractions.

bioRxiv preprint doi: https://doi.org/10.1101/2024.02.13.580128; this version posted January 14, 2025. The copyright holder for this preprint (which was not certified by peer review) is the author/funder, who has granted bioRxiv a license to display the preprint in perpetuity. It is made available under aCC-BY 4.0 International license.

Supplemental Table 1. Cryo-EM data collection, refinement, and validation statistic.

	GmCesA1 (EMDB-43244) (PDB: 8VHZ)	GmCesA3 (EMDB-43241) (PDB: 8VHT)	GmCesA6 (EMDB-43245) (PDB 8VI0)
**Data collection and processing**			
Microscope	Titan Krios	Titan Krios	Titan Krios
Camera	GIF K3	GIF K3	GIF K3
Magnification	81,000	81,000	81,000
Voltage (kV)	300	300	300
Electron exposure (e–/Å^2)	50	50	50
Defocus range (µm)	-1.1 to -2.2	-1.1 to -2.2	-1.1 to -2.2
Pixel size (Å)	1.08	1.08	1.08
Symmetry imposed	C3	C3	C3
Initial particle images (no.)			
Final particle images (no.)	181,101	237,568	192,666
Map resolution (Å)	3.3	3.2	3.0
FSC threshold	0.143	0.143	0.143
Map resolution range (Å)	3.3 -	3.2 -	3.0 -
**Refinement**			
Initial model used	AlphaFold generated	AlphaFold generated	AlphaFold generated
Map sharpening *B* factor (Å^2)	-117.5	-105.1	-92.4
Model composition			
Non-hydrogen atoms	17190	16644	17694
Protein residues	2139	2064	2172
Ligand		BGC	BGC
Mean *B* factors (Å^2)			
Protein	39.47	56.40	38.03
Ligand		85.27	72.40
R.m.s. deviations			
Bond lengths (Å)	0.003	0.002	0.002
Bond angles (°)	0.603	0.53	0.499
Ramachandran plot			
Favored (%)	96.88	97.05	97.7
Allowed (%)	3.12	2.95	2.3
Disallowed (%)	0.00	0.00	0.00
Validation			
MolProbity score	1.52	1.42	1.58
Clashscore	6.32	4.91	6.87
Poor rotamers (%)	0.16	0.00	0.00
Overall correlation coefficients			
CC (mask)	0.76	0.82	0.85
CC (peaks)	0.62	0.79	0.74
CC (volume)	0.74	0.66	0.83
